# Preparation of 2'-^13^C-L-Histidine Starting from ^13^C-Thiocyanate: Synthetic Access to Any Site-Directed Stable Isotope Enriched L-Histidine

**DOI:** 10.3390/molecules19011023

**Published:** 2014-01-15

**Authors:** Sarra Talab, Kamal Khalifa Taha, Johan Lugtenburg

**Affiliations:** 1 Department of Chemistry, College of Applied and Industrial Science, University of Bahri, P.O. Box 11111, Khartoum 1660, Sudan; 2 Department of Chemistry, College of Science, AI Imam Mohammad Ibn Saud Islamic University(IMSIU), P.O. Box 5701, Riyadh 11432, Kingdom of Saudi Arabia; E-Mail: kamaltha99@rediffmail.com; 3 Leiden Institute of Chemistry, Leiden University, P.O. Box 9502, 2300 RA Leiden, The Netherlands; E-Mail: lugtenbu@chem.leidenuniv.nl

**Keywords:** 1-benzyl-2-(methylthio)-5-imidazole carbonitrile, ethyl-1-benzyl-5-imidazol-carboxylate, *N*-diphenylmethylene glycine *tert*-butyl ester, O’Donnell method

## Abstract

1-Benzyl-2-(methylthio)-imidazole-5-ketone is obtained in a few simple steps starting from thiocyanate and glycine amide (glycin). Subsequent treatment with diethyl phosphorocyanidate and functional group manipulations gives 1-benzyl-5-chloromethyl-imidazolium chloride. This compound is converted under mild O’Donnell conditions into the corresponding L-histidine derivative. After deprotection L-histidine is obtained in good yield and 99% enantiomeric excess. 2'-^13^C-L-Histidine has been obtained via this new scheme with high (99%) ^13^C incorporation starting with commercially available ^13^C- thiocyanate. This synthetic scheme allows access to any isotopomer of L-histidine and many other biologically important imidazole derivatives.

## 1. Introduction

L-Histidine constitutes a unique amino acid residue in peptides and proteins. Its pK_a_ value is in the range of 6.4–7.0, so histidine residues account to a large extent for the buffer capacity of the protein system [1]. The histidine links with the central metal in haemoglobin, myoglobin, cytochrome and (bacterio) chlorophyll and binds with other metal ions [2]. Via its role in the above mentioned systems it is intimately linked to vital life processes such as respiration and photosynthesis. The access to a whole library of side directed stable isotope-enriched histidine is essential to study the role of histidine in the proteins which are responsible for respiration and photosynthesis at the atomic level with isotope sensitive techniques without perturbation [3]. This deeper level of knowledge obtained in this way is essential to get a fundamental understanding of these important life processes. In this paper we describe the synthesis of 2'-^13^C-L-histidine (**1a**) via a new scheme that also allows access to any isotopologue and isotopomer of L-histidine. In the present scheme the O’Donnell asymmetric amino acid synthesis is used, allowing mild reaction conditions and simple workups [4]. In the previous schemes preparative HPLC techniques had to be used [5,6]. Benzyl isothiocyanate is formed in a simple reaction between potassium thiocyanate and benzyl chloride.

## 2. Results and Discussion

**Scheme 1:**
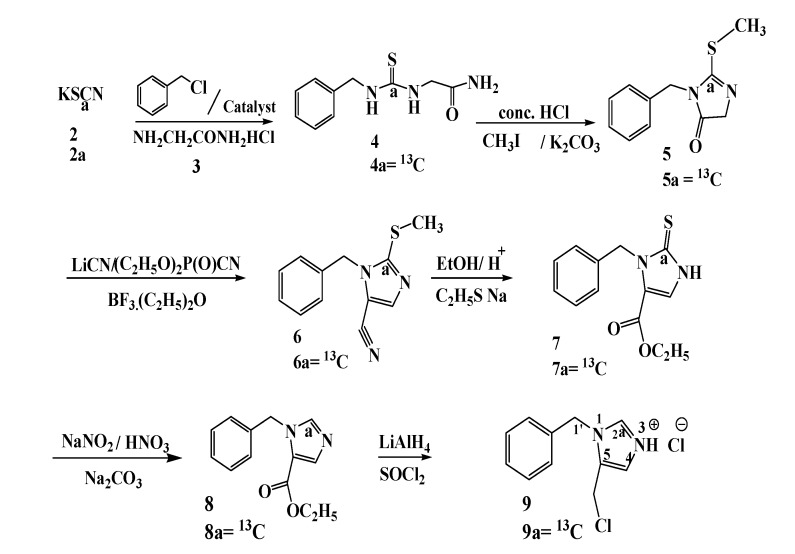
The preparation of 1-benzyl-5-chloromethylimidazolium chloride **9** starting from potassium thiocyanate **2** giving stable isotope incorporation in **9** at position 1 and/or 2.

For the access to stable isotope labeled 2-^13^C 1-benzyl-5-chloromethylimidazolium chloride (**9a**) the reactions depicted in Scheme 1 were optimized with natural abundance reagents. Potassium thiocyanate (**2**) is reacted with benzyl chloride in o-dichlorobenzene in the presence of the phase transfer catalyst bis(triphenyl)phosphoranylidene ammonium chloride. According to a known procedure a mixture containing mainly benzyl isothiocyanate and some benzyl thiocyanate is obtained [7]. This isomeric mixture is treated with glycine amide HCl **3 ** at pH 8.5 [8]. The product is recrystallized from acetone and benzyl thiourea **4** is thus obtained.

For the cyclisation of **4** it is dissolved in acetone and aqueous HCl [9]. When the reaction is finished the 1-benzyl-2-thioxoimidazolium-4-one is obtained by extraction (not shown in Scheme 1). The conversion of benzyl isothiocyanate and 1-benzyl-2-thioxoimidazolium-4-one is analogous to the reaction that takes place in the Edman sequencing [10] of proteins and is a high yielding, efficient process. The conversion can also be effected with glycine instead of **3** [8].

1-Benzyl-2-thioxoimidazolium-4-one dissolved in acetonitrile is treated with methyl iodide in the presence of potassium carbonate under known conditions to form 1-benzyl-2-(methylthio)-imidazol-5-ketone (**5**) [10]. A conversion of aromatic ketones into unsaturated nitriles has been described [11].

We hoped that the amide function of **5** would undergo a similar conversion with diethyl phosphoro-cyanidate. Compound **5** is treated with diethyl phosphorocyanidate in the presence of LiCN in THF. After evaporation of THF the product extracted with ethyl acetate is the cyanohydrin phosphate corresponding to **5**. Treatment of this product in benzene with boron trifluoride etherate results in the elimination of diethyl phosphate giving the aromatic nitrile **6**, which is then dissolved in ethanol and after the addition of concentrated sulphuric acid, the solution is refluxed. After the workup crystalline ethyl-1-benzyl-2-ethyl thio-5-imidazole carboxylate is obtained (not shown in Scheme 1).

During the reaction of **6** with acidified ethanol not only the expected conversion of the nitrile function into the ethyl carboxyl function, but also acid catalyzed conversion of the methylthio group into an ethylthio function had taken place. This simple conversion of **6** into ethyl-1-benzyl-2-ethyl thio-5-imidazole carboxylate was found after failed attempts of DIBAL-H reduction and Raney nickel desulphurization of **6**.

Ethyl-1-benzyl-2-ethyl thio-5-imidazole carboxylate is treated with sodium ethylthiolate in DMF [12]. This gives ethyl-1-benzyl-2,3-dihydro-2-thioxo-5-imidazole-carboxylate **7** after crystallization. This compound was an intermediate in our earlier preparation of [1'-5' N] and [3'-5' N] L-histidine [6] and can be converted into 1-benzyl-5-chloromethylimidazolium chloride (**9)**.

**Scheme 2:**
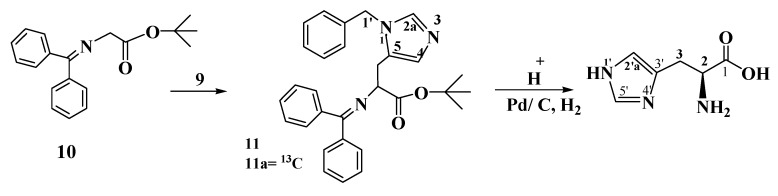
The conversion of 1-benzyl-5-chloromethylimidazolium chloride (**9**) under O’Donnell conditions into the protected L-histidine compound **11**. Final deprotection leads to stable isotope enriched L-histidine (**1a**).

The imidazolium chloride salt **9** is treated according to Scheme 2 with *tert*-butyl(N-diphenyl methylene) glycinate (**10**) under O’Donnell conditions [4]. The protected histidine derivative **11** is obtained in high yield. Acid catalysis removes the *tert*- butyl ester and the amino protection. A final hydrogenlysis with palladium on activated charcoal gives L-histidine (**1**) with 99% chemical purity and 99% optical purity (via HPLC as described in the Experimental).

Compound **7** is treated briefly with NaNO_2_/HNO_3_ giving desulphurization to 1-benzyl-5-carbo-ethoxyimidazole (**8**). Subsequently reduction of **8** with LiAlH_4_ converts the ethyl function into the methylene alcohol function. Treatment of the latter product with SOCl_2_ gives the imidazolium chloride salt **9**. The analytical data of **9** are within experimental error in agreement with those reported by us before [6].

## 3. Experimental

### 3.1. General

All chemicals were purchased from Sigma-Aldrich (Zwijndrecht, The Netherlands) or Acros Chimica (Geel, Belgium). In all experiments anhydrous solvents were used. Ether refers to diethyl ether and all solvent mixtures are given in volume ratio (v/v). All solvents were removed by evaporation *in vacuo*.

Reactions were monitored by thin-layer chromatography [TLC, on Merck F 254 silica gel 60 aluminum sheets, 0.2 mm: spots were visualized by treatment with an oxidizing spray (2 g of KMnO_4_ and 4 g of NaHCO_3_ in 100 mL of water)]. Column chromatography was performed on Merck silica gel 60. ^1^H- and ^13^C spectra were recorded with a Bruker WM-300 or WM-400 with tetramethylsilane (TMS: δ 0.00 ppm) as internal standard for spectra recorded in CDCl_3_. A Perkin-Elmer Paragon 1000 FTIR spectrometer and Varian Cary 50 series UV spectrometer were used for IR and UV measurement, respectively.

The optical purities of the histidines were determined by HPLC and are given as molar fractions (%). The apparatus consisted of a Spherisorb ODS-2 (5 μm) column (250 × 4 mm), a Pharmacia LKB gradient delivery system, a Reheodyne injection valve fitted with a 5-μL loop, and a Spectro Vision FD-300 fluorescence detector. Prior to chromatography the histidines were derivatized using *o*-phthalaldehyde (OPA, Janssen, vosselaar Beerse, Belgium) and *N*-acetyl-L-cysteine (NAC, Aldrich) [13]. The excitation and emission wavelengths were 360 and 405 nm, respectively. Derivatization was carried out using a solution of 2 mg of histidine in H_2_O (I), a 0.2 M borax buffer (pH 10.4) (II), a solution of 10 mg of OPA in 5 mL of methanol (III) and a solution of 10 mg of NAC in 5 mL of methanol (IV). 10 μL of I, 20 μL of II, 10 μL of III and 10 μL of IV were added together. The mixture was shaken for 2 min and injected. The diastereomers were separated employing a linear elution gradient of 100% buffer A2 (30 mM sodium acetate pH 4) to 80% buffer A2 and 20% buffer B2 (50% 30 mM sodium acetate pH 7.6/50% acetonitrile) in 60 min (0.4 mL/min). The retention time was 50 min.

Mass spectra were recorded on a Finnigan MAT-90 mass spectrometer (CI-MS, CH_4_) or on a Finnigan MAT ITD 700 mass spectrometer (EI, 70 eV) coupled to a Hewlett Packard 438 A gas chromatograph equipped with a Hewlett Packard 25-m SE 30 capillary column (GC-MS). Prior to EI-MS and CI-MS the amino acids were derivatized to their corresponding *n*-butyl esters. The sample was treated with 3 M HCl in *n-*BuOH for 15 min at 100 °C under an anhydrous nitrogen atmosphere in a sealed Kimax tube. The solvent was evaporated and the sample was injected as a solution in dichloromethane. The procedures used for the preparation for the preparation of the isotopically enriched compound were the same as those described for the corresponding unlabelled compounds. Only spectroscopic characteristics of the enriched compounds that differ from those of the unlabelled compound are given.

### 3.2. Preparation of Compounds

*N-Benzyl-N'-(2'-acetamido) thiourea* (**4**). First benzyl isothiocyanate is prepared. Benzyl chloride (3.0 g, 23.7 mmol, 2.27 mL) is dissolved in *o*-dichlorobenzene (10 mL) to which the phase transfer catalyst bis(triphenyl) phosphoranylidene ammonium chloride (0.57 g, 0.5 mmol) and solid potassium thiocyanate (**2**, 2.5 g, 25.7 mmol) is added. The mixture is heated under reflux for three hours at 180 °C. After cooling to room temperature the solvent is removed under reduced pressure, the residue is extracted with chloroform (3 × 10 mL). The chloroform solution is washed with water and then dried over MgSO_4_. After drying the solvent is removed under reduced pressure to yield benzyl isothiocyanate as a yellow oil 2.9 g, 19.46 mmol (82.1%). ^1^H-NMR spectroscopy shows that the product consists of a major compound (benzyl isothiocyanate , 83.0%) and a minor compound (benzyl thiocyanate, 17.0%). *Benzyl isothiocyanate*: ^1^H-NMR (300 MHz, DMSO, δ in ppm): 4.91 (2H, benzyl CH_2_), 7.31–7.42 (5H, aromatic CH); and (2) ^13^C-NMR (75.5 MHz, DMSO, δ in ppm): 47.9 (benzyl CH_2_), 127.0 (CH_Ph_), 128.0 (CH_Ph_), 128.5 (CH_Ph_), 129.2 (CS), 134.6 (Cq). *Benzyl thiocyanate*: ^1^H-NMR (300 MHz, DMSO, δ in ppm): 4.34 (2H, benzyl CH_2_), 7.31–7.42 (5H, aromatic CH) and (2) ^13^C-NMR (75.5 MHz, DMSO, δ in ppm): 37.0 (benzyl CH_2_), 127.0 (CH_Ph_), 128.0 (CH_Ph_), 128.5 (CH_Ph_), 129.2 (CS), 133.5 (Cq).

Glycinamide hydrochloride (**3**, 4.0 g, 36.1 mmol) is dissolved in water (24 mL). To this mixture a 50% solution of KOH (35.7 mmol, 2 g in 3.0 mL of water) is added to adjust the pH to 8.5, and then ethanol (72.0 mL) is added. The solution is cooled in ice bath to 0 °C, and then treated dropwise with the previously prepared mixture containing 83.0% benzyl isothiocyanate and 17.0% benzyl thiocyanate (5.39 g, 4.7 mL, 36.1 mmol). The reaction mixture is stirred for 3 h, and frozen at −30 °C overnight. The mixture is allowed to reach room temperature, the solvent is decanted from the solid compound, and the solid compound is dried *in vacuo* to remove the remaining ethanol and water. The product is recrystallized from acetone to afford 7.0 g (31.4 mmol, 86.98%) of *N*-benzyl-*N'*-(2'-acetylamido) thiourea as pale yellow crystals. ^1^H-NMR (300 MHz, DMSO, δ in ppm): 4.04 (2H, C-5), 4.66 (2H, C-1), 7.32 (5H, aromatic CH), 8.30 (1 H, N-2), 7.54 (1H, N-4), 7.11 (2H, N-7); ^13^C-NMR (75.5 MHz, DMSO, δ in ppm): 42.3 (C-5), 45.1 (C-1), 126.8, 127.2, 128.2, 134.19 (Cq), 170.5 (C6, Cq), 175.0 (C3, Cq).

*[^13^C] N-Benzyl-N'-(2'-acetamido) thiourea* (**4a**). First ^13^C benzyl isothiocyanate (1.2 g, 8.0 mmol) is prepared (80.0% yield) as described above from KS^13^CN (1.0 g, 10.3 mmol). ^1^H-NMR: the signal at 4.91 ppm was split into a doublet (^3^J_C-H1_ = 2.6 Hz).^13^C-NMR: enhanced signal at 129.2 ppm. Then [13C]-N-benzyl-N'-(2'-acetamido)thiourea (**4a**, 1.5 g, 6.5 mmol) is prepared in 89.04% yield from ^13^C-benzyl isothiocyanate (1.1 g, 7.3 mmol) as described for **4**. ^1^H-NMR: the signals at 4.11 and 4.65 were split into doublets (^3^J_C3H5_ = 4.8 Hz, ^3^J_C3H1_ = 5.6 Hz); ^13^C-NMR: enhanced signal at 175.0 ppm.

1*-Benzyl-2-(methylthio)-imidazol-5-ketone* (**5**). Benzyl thiourea (**4**, 7.0 g, 31.3 mmol) is dissolved in acetone (25 mL) and treated carefully with concentrated HCl (3 mL for each gram of compound) and the mixture is then heated overnight in an oil bath at 40 °C (the reaction is followed with TLC until the starting compound has disappeared). The acetone is removed *in vacuo* and the residue is treated with sat. aq. NaHCO_3_ to neutralize the excess acid and the mixture is extracted three times with CH_2_Cl_2_. Then the combined organic layers are washed with water and brine (100 mL), the dichloromethane layer is dried with MgSO_4_, filtered and concentrated *in vacuo*. The product is purified on a column of silica gel (dichloromethane/methanol = 95/5) to give a yellow crystalline compound (5.05 g, 78.1%). This compound (3-benzyl-2-thioimidazolidin-4-one) is dissolved in acetonitrile (30 mL). To this mixture iodomethane (5.24 g, 36.9 mmol) and potassium carbonate (1.7 g, 12.3 mmol) are added. The mixture is stirred at 40 °C for 14 h. The reaction mixture is cooled to room temperature and then filtered. The solvents are removed *in vacuo*. The residue is dissolved in a mixture of dichloromethane and hexane (1:1, 50 mL), filtered and the solvents are removed *in vacuo*. The product is purified on a column of silica gel (dichloromethane/methanol = 97/3) yielding 1-benzyl-2-(methylthio)-imidazol-5-ketone as a green crystalline compound (5.15 g, 23.4 mmol, 75%). ^1^H-NMR (300 MHz, DMSO, δ in ppm): 2.22 (3H, methyl thiogroup CH_3_), 2.99 (2H, 5-CH_2_), 4.65 (2H, benzyl CH_2_), 7.21–7.33 (5H, aromatic CH); ^13^C-NMR (75.5 MHz, DMSO, δ in ppm): 12.0 (CH_3_), 43.0 (C-5), 58.3 (benzyl CH_2_), 127.0, 127.6, 128.5, 136.1 (Cq), 162.1 (C2), 179.8 (C4, Cq).

*[^13^C] 1-Benzyl-2-(methylthio)-imidazol-5-ketone* (**5a**). ^13^C-1-benzyl-2-(methylthio)-imidazol-5-ketone (**5a**, *1*.25 g, 5.6 mmol, 96.5% yield) is prepared as described for **5** from ^13^C-benzylthiourea (1.3 g, 5.8 mmol). ^1^H-NMR: the signals at 2.22 and 4.65 ppm were split into doublets (^3^J_C2H5_ = 4.8 Hz, ^3^J_C2H3'_ = 5.6 Hz); ^13^C-NMR: enhanced signal at 162.1 ppm.

*1-Benzyl-2-(methylthio)-5-imidazolcarbonitrile* (**6**). 1-Benzyl-2-(methylthio)-imidazol-5- ketone (**5**, 3 g, 13.6 mmol) is dissolved in THF (130 mL), and to this mixture diethyl phosphorocyanidate (DEPC, 6.67 g, 40.9 mmol) and lithium cyanide (1.3 g, 39.3 mmol) are added gradually [the lithium cyanide is prepared by using a 250 mL round bottomed flask equipped with a magnetic stirrer, nitrogen inlet and a 60 mL addition funnel; lithium hydride (1.0 g, 126 mmol) is dissolved in anhydrous tetrahydrofuran (100 mL). The stirred suspension is cooled in an ice bath and acetone cyanohydrin (9.14 g, 9.14 mL, 100 mmol) is added dropwise over 15 min. After the addition is completed, the ice bath is removed and the mixture stirred for 2 h at room temperature. The magnetic stirring bar is removed and the solvent evaporated *in vacuo* to yield lithium cyanide as a white powder (4.07 g, 123.4 mmol, 99.2%)].

After the addition of LiCN is completed the reaction mixture is stirred at room temperature for 10 min. After removal of THF by evaporation the residue is dissolved in water (130 mL) and a mixture of benzene/ethyl acetate (1/1, 400 mL). The organic layer is separated and washed with water (2 × 100 mL) and saturated aqueous sodium chloride (1 × 100 mL). The organic layer is separated and then dried over MgSO_4,_ followed by concentration to give green oil, which is dissolved directly in benzene (20 mL) followed by addition of boron trifluoride etherate (5.8 g, 40.9 mmol). The reaction mixture is stirred at room temperature for 2 h under nitrogen. After 2 h benzene (200 mL) and water (40 mL) are added to the reaction mixture, the organic layer is separated, and washed with water (2 × 100 mL) and saturated aqueous sodium chloride (150 mL). The organic layer is separated, dried over MgSO_4_ followed by concentration giving a brown oil of the nitrile, which is purified on a silica gel column (CH_2_Cl_2_/MeOH = 95/5). The result is 2.3 g (10.04 mmol, 73.8%) of the desired compound **6** as a brown oil. ^1^H-NMR (300 MHz, DMSO, δ in ppm): 2.49 (3H, CH_3_), 5.15 (2H, benzyl CH_2_), 6.92 (H, C4), 7.16 (2H, aromatic CH), 7.41 (3H, aromatic CH); ^13^C-NMR (75.5 MHz, DMSO, δ in ppm): 15.9 (methyl thio group CH_3_), 46.1 (benzyl CH_2_), 104.1 (C5), 111.9 (CN), 126.5, 127.1, 128.6, 136.1 (Cq), 136.4 (C4, Cq), 138.0 (C2).

*[^13^C] 1-Benzyl-2-(methylthio)-5-imidazol carbonitrile* (**6a**). [^13^C]-1-benzyl-2-(methylthio)-5-imidazolcarbonitrile (**6a**, 1.05 g, 3.8 mmol, 70.4% yield) is prepared as described for **6** from ^13^C-1-benzyl-2-(methylthio)-imidazol-5-ketone (1.2 g, 5.4 mmol). ^1^H-NMR: the signals at 2.49 and 5.15 ppm were split into doublets (^3^J_C2H5_ = 4.6 Hz, ^3^J_C2H3'_ = 5.6 Hz); ^13^C-NMR: enhanced signal at 138.0 ppm.

*Ethyl 1-benzyl-2,3-dihydro-2-thioxo-5-imidazole-carboxylate* (**7**). 1-Benzyl-2-(methylthio)-5-imidazol-carbonitrile (**6**, 1.5 g, 6.5 mmol) is dissolved in ethanol (10 mL), the mixture is cooled to 0 °C in an ice bath and 0.53 mL of concentrated sulfuric acid (9.825 mmol) is added dropwise and carefully. After the addition is completed the ice bath is removed and the mixture is heated under reflux for 24 h. The mixture is cooled to room temperature and poured into a flask containing ice water (50 mL); the pH is adjusted to 3 with an aqueous solution of KOH. The mixture is filtered and the filtrate is concentrated *in vacuo* to yield a yellow solid of the desired compound, the solid is recrystallized from dichloromethane to give brown needles (1.7 g, 5.86 mmol, 89.9%).

The latter is added to a suspension of sodium ethanethiolate (10.6 mmol, 0.85 g) in DMF (8 mL). The mixture is heated at reflux overnight; the DMF is removed *in vacuo* to afford a crude solid. The solid is dissolved in a minimal amount of ethanol (12 mL), and then silica gel (5 g) is added to this mixture. The mixture is filtered carefully and washed with ethanol (5 mL). The residue is treated with a mixture of CH_2_Cl_2_/EtOH = 95/5 to yield a yellow oil (1.29 g, 4.94 mmol, 76.0%) which is used directly in the next step.

*[^13^C] Ethyl 1-benzyl-2,3-dihydro-2-thioxo-5-imidazolecarboxylate* (**7a**). ^13^C Ethyl 1-benzyl-2,3-dihydro-2-thioxo-5-imidazole-carboxylate (**7a**, 1.08 g, 4.1 mmol, 95.3% yield) is prepared as described for **7** from ^13^C 1-benzyl-2-(methylthio)-5-imidazolcarbonitrile (1.0 g, 4.3 mmol).

*1-Benzyl 5-carbo-ethoxy imidazole* (**8**). The yellow compound **7** is dissolved in a NaNO_2_ solution (1.0 mL, 0.5 g NaNO_2_ in 237 mL of H_2_O) and then nitric acid (65%, 0.35 mL) is added with continuous stirring. The mixture is stirred for 30 min. and then boiled with a solution of sodium carbonate (40% in water, 8 mL). The product is extracted with ether, dried over MgSO_4_ and concentrated. Purification is effected by column chromatography (CH_2_Cl_2_/EtOH = 97/3) to yield a brown oil (950 mg, 4.13 mmol, 83.6%). ^1^H-NMR (400 MHz, DMSO, δ in ppm): 1.19 (3H, ethyl ester group CH_3_), 3.94 (2H, ethyl ester group CH_2_), 4.45 (2H, benzyl CH_2_), 6.75 (1H, C5), 7.19–7.35 (5H, aromatic CH), 8.13 (1H, C2); ^13^C-NMR (100 MHz, DMSO, δ in ppm): 16.1 (CH_3_), 42.9 (CH_2_), 43.4 (benzyl CH_2_), 119.2 (C5), 126.2, 126.7, 127.9, 138.8 (Cq), 134.5 (C4, Cq), 141.2 (C2), 158.2 (CO).

*[^13^C] 1-Benzyl 5-carboethoxy imidazole* (**8a**). ^13^C 1-Benzyl-5-carboethoxy imidazole (**8a**, 750 mg, 3.2 mmol, 88.9% yield) is prepared as described for **8** from ^13^C ethyl 1-benzyl-2,3-dihydro-2-thioxo-5-imidazolecarboxylate (950 mg, 3.6 mmol). ^1^H-NMR: the signals at 4.45, 6.75 and 8.13 ppm were split into doublets (^3^J_C2H5_ = 4.8 Hz, ^3^J_C2H3'_ = 5.6 Hz, ^1^J_C2H2_ = 219 Hz); ^13^C-NMR: enhanced signal at 141.2 ppm.

*1-Benzyl-5-chloromethylimidazolium chloride* (**9**). In a 100 mL three-necked flask lithium aluminum hydride (0.16 g, 4.4 mmol) is suspended in THF (20 mL) under a dry argon atmosphere. To this suspension a solution of 1-benzyl 5carboethoxy imidazole (**8**, 930 mg, 3.5 mmol) in THF (15 mL) is added dropwise with continuous stirring (15 min). The reaction is followed by TLC until the latter has disappeared (2.5 h, TLC: CH_2_Cl_2_/EtOH = 95/5). The mixture is carefully diluted with water (12 mL) and subsequently with 6 M HCl (12 mL). The pH is adjusted to 9 using a 6 M NaOH solution and the aqueous layer is extracted with dichloromethane. The organic layers are combined, washed with saturated aqueous NaCl and dried over anhydrous MgSO_4_. After filtration the solvent is removed *in vacuo*, yielding a yellow oil (660 mg, 3.19 mmol, 86.8%). A portion (500 mg) of this compound is placed in a 100 mL round bottomed flask fitted with a reflux condenser to which thionyl chloride (1.23 g, 10.0 mmol) is added. The mixture is refluxed for three h, and then allowed to cool to room temperature. The volatiles are evaporated *in vacuo*. The result is a brown oil (560 mg, 2.45 mmol, 70.0%) of the desired compound. ^1^H-NMR (400 MHz, CDCl_3_, δ in ppm): 4.36 (2H, CH_2_Cl), 4.69 (2H, benzyl CH_2_), 6.90 (1H, C5), 7.31–7.40 (5H, aromatic CH), 7.91 (1H, C2); ^13^C-NMR (100 MHz, CDCl_3, _δ in ppm): 44.6 (C-4'), 52.2 (benzyl CH_2_), 111.7 (C5), 127.5, 127.7, 129.0, 131.9 (C2), 135.6 (Cq), 144.0 (C4, Cq).

*[^13^C] 1-Benzyl-5-chloromethylimidazolium chloride* (**9a**). ^13^C 1-benzyl-5-chloromethylimidazolium chloride (**9a**, 540 mg, 2.21 mmol, 73.7% yield) is prepared as described for **9** from ^13^C 1-benzyl-5-carboethoxyimidazole (700 mg, 3.0 mmol). ^1^H-NMR: the signals at 4.69, 6.90 and 7.91 were split into doublets (^3^J_C2H5_ = 4.6 Hz, ^3^J_C2H3'_ = 5.6 Hz, ^1^J_C2H2_ = 221 Hz). ^13^C-NMR: enhanced signal at 131.9 ppm.

*Tert-butyl-3-(1-benzyl-1H-imidazol-5-yl)-2(diphenylmethyleneamino) propanoate* (**11**). N-(Diphenyl-methylene) glycine tert-butyl ester (**10**, 570 mg, 1.92 mmol) and the chiral catalyst 2,7-bis [O-9-allyl hydrocinchonidinium *N*-methyl]naphthalene dibromide (19 mg, 0.0019 mmol) are dissolved in toluene (10 mL). To this mixture 1-benzyl-5-chloromethylimidazolium chloride (**9**, 500 mg, 2.05 mmol) is added. The reaction mixture is then cooled to 0 °C, and 50% aqueous KOH (0.28 mL) is added, the mixture is stirred at 0 °C until the starting material has been consumed (8 h). The ice bath is removed and the mixture is diluted with ether (25 mL), washed with water (2 × 15 mL), the ether layer is separated, dried over MgSO_4_, filtered and concentrated to yield a brown crystalline compound (780 mg, 1.67 mmol, 87.5% yield). ^1^H-NMR (300 MHz, DMSO, δ in ppm): 1.39 (9H, CH_3_), 4.03 (2H, benzyl CH_2_), 4.48 (2H, CH_2_), 4.94 (1H, C3'), 6.90 (1H, C5), 7.1–7.55 (15H, aromatic CH), 7.90 (1H, C2); ^13^C-NMR (100 MHz, DMSO_, _δ in ppm): 27.6 (3C, CH_3_), 53.3 (C-4'), 55.7 (benzyl CH_2_), 67.2(C, C3'), 75.6 (C, OMe_3_), 125.2 (C5), 126.9–128.8 (15 C, CH_Ph_), 135.0 (C4_, _Cq), 135.0 (C2), 137.3–138.6 (Cq), 168.9 (C=N), 170.0 (CO).

*[^13^C] Tert-butyl-3-(1-benzyl-1H-imidazol-5-yl)-2(diphenylmethyleneamino) propanoate* (**11a**). ^13^C tert-Butyl-3-(1-benzyl-1H-imidazol-5-yl)-2(diphenylmethyleneamino) propanoate (**11a**, 1.1 g, 2.36 mmol, 95.9% yield) is prepared as described for **11** from ^13^C 1-benzyl-5-chloromethylimidazolium chloride (600 mg, 2.46 mmol). ^1^H-NMR: the signals at 4.03, 6.90 and 7.90 were split into doublets (^3^J_C2H5_ = 4.8 Hz, ^3^J_C2H1'_ = 5.6 Hz, ^1^J_C2H2_ = 220 Hz). ^13^C-NMR: enhanced signal at 135.0 ppm.

L*-Histidine dihydrochloride* (**1**). *tert*-Butyl-3-(1-benzyl-1*H*-imidazol-5-yl)-2-(diphenylmethylene-amino) propanoate (**11**, 400 mg, 0.86 mmol) is dissolved in THF (10 mL) and then stirred with 10% citric acid solution (5 mL) overnight. TLC shows complete disappearance of the starting material. The mixture is extracted twice with ether (20 mL). The water layer is then brought to pH 12 with a K_2_CO_3_ solution and extracted with ethyl acetate (10 mL). The combined organic layers are dried over MgSO_4_ and concentrated *in vacuo* to give brown crystals (250 mg, 0.828 mmol, 96.3%). This compound is then treated with 1 N HCl (5 mL), and the mixture is refluxed overnight. The reaction is followed by TLC until the starting material has disappeared (dichloromethane/ethanol = 99/1). The mixture is allowed to reach room temperature, diluted with a small amount of dichloromethane, filtered and concentrated *in vacuo*. The product is a yellow oil (230 mg, 0.709 mmol, 85.7%). The latter is dissolved in a mixture of cyclohexene/methanol (1/1). Palladium black (100 mg) is added to the mixture. The mixture is refluxed until the conversion is complete, which takes 5 days. On the second day a freshly prepared Pd pellet [6] (50 mg in 10 mL of the same solvent mixture) is added to the mixture. The suspension is cooled down to room temperature and filtered over Celite. The solvent is evaporated *in vacuo*; the compound is redissolved in methanol and then concentrated to yield a yellow solid (165 mg, 0.727 mmol, 84.5%). ^1^H-NMR (400 MHz, D_2_O/DCl, δ in ppm): 3.46 (1H, H3a), 3.62 (1H, H3b), 4.42 (1H, H2), 7.49 (1H, H5'), 8.74 (1H, H2'); ^13^C-NMR (100 MHz, D_2_O/DCl, δ in ppm): 27.9 (C3),54.7 (C2), 118.5 (C5'), 130.0 (C4'), 135.8 (C2'), 174.0 (C1).

*2'-[^13^C]-L-Histidine dihydrochloride* (**1a**). 2'-^13^C-*L*-Histidine dihydrochloride (**1a**, 390 mg, 1.7 mmol, 80.9% yield) is prepared as described for **1** from ^13^C tert-butyl-3-(1-benzyl-1H-imidazol-5-yl)-2-(diphenylmethyleneamino) propanoate (1.0 g, 2.1 mmol). ^1^H-NMR: the signals at 7.49 and 8.74 ppm were split into doublets (^3^J_C2H5'_ = 6.5 Hz, ^1^J_C2'H2'_ = 221 Hz). ^13^C-NMR: enhanced signal at 135.8 ppm. Both chemical and optical purity is 99% as found by an HPLC method, in agreement with the literature [5]. The more volatile n-butyl ester of 2'-^13^C-L-histidine was investigated by double focus chemical ionization mass spectroscopy (ionization gas CH_4_). The signal at *m/z* = 213.1439 corresponds with ^12^C_9_^13^C^1^H_18_^14^N_3_^16^O_2_ (calculated mass: 213.1433). In the single focus EI/70 eV mass spectrum the fragment at *m/z* = 82 is formed by McLafferty rearrangement involving the imidazole ring. From the base peak and the M-1, M+1 and M+2 peaks 99% enrichment with ^13^C at the C2' could be established within experimental error and in agreement with the ^13^C incorporation in ^13^C thiocyanate. No isotope dilution has taken place during the reaction sequence.

## 4. Conclusions

A simple procedure to obtain 2'-^13^C-L-histidine in high optical purity and high ^13^C incorporation is described. The analytical properties of 2'-13C-L-histidine are within experimental error in agreement with the literature [5]. The building blocks that form the imidazole ring and L-histidine in Scheme 1 and Scheme 2 are either commercially available or easily accessible via literature procedures [3]. This means that L-histidine is accessible in any stable isotopologic and isotopomeric form. Using the optical antipode of the O’Donnell phase transfer catalyst will give access to all possible D-histidines in isotopomeric form. Recently the simple incorporation of ^2^H and ^3^H in histidine has been reported [14], which together with the work described in this paper extends the access to stable and unstable isotopomers and isotopologues of histidine considerably.
